# Sodium-Glucose Cotransporter-2 Inhibitors and Cardiorenal Events in Nonalbuminuric Kidney Disease

**DOI:** 10.1016/j.ekir.2025.103756

**Published:** 2025-12-30

**Authors:** Fu-Shun Yen, Keong Chong, Chien-Wei Huang, James Cheng-Chung Wei, Jia-Sin Liu, Yi-Ling Wu, Chih-Ming Chen, Chii-Min Hwu, Chih-Cheng Hsu

**Affiliations:** 1 Dr. Yen’s Clinic, Taoyuan, Taiwan; 2 Department of Endocrinology, Min-Sheng General Hospital, Taoyuan, Taiwan; 3 Division of Nephrology, Department of Internal Medicine, Kaohsiung Veterans General Hospital, Kaohsiung, Taiwan; 4 School of Medicine, National Yang Ming Chiao Tung University, Taipei, Taiwan; 5 Department of Allergy, Immunology and Rheumatology, Chung Shan Medical University Hospital, Taichung City, Taiwan; 6 Institute of Medicine, Chung Shan Medical University, Taichung City, Taiwan; 7 Graduate Institute of Integrated Medicine, China Medical University, Taichung, Taiwan; 8 Institute of Population Health Sciences, National Health Research Institutes, Miaoli County, Taiwan; 9 Faculty of Medicine, National Yang Ming Chiao Tung University School of Medicine, Taipei, Taiwan; 10 Section of Endocrinology and Metabolism, Department of Medicine, Taipei Veterans General Hospital, Taipei, Taiwan; 11 Department of Health Services Administration, China Medical University, Taichung, Taiwan; 12 National Center for Geriatrics and Welfare Research, National Health Research Institutes, Yunlin County, Taiwan; 13 Department of Family Medicine, Min-Sheng General Hospital, Taoyuan, Taiwan

**Keywords:** cardiorenal outcomes, nonalbuminuric kidney disease, population-based cohort study, sodium-glucose cotransporter-2 inhibitors, type 2 DM

## Abstract

**Introduction:**

Most patients with type 2 diabetes (T2D) and chronic kidney disease (CKD) do not exhibit albuminuria. However, data are limited regarding the potential benefits of sodium-glucose cotransporter-2 inhibitors (SGLT2is) in this subgroup.

**Methods:**

We conducted a population-based cohort study between May 1, 2016, and December 31, 2021 using Taiwan’s National Health Insurance (NHI) Research Database to examine the association between SGLT2is use and outcome in patients with T2D and nonalbuminuric CKD. Cox proportional hazard models were used to determine the risk of outcomes between the study and control groups.

**Results:**

The study follow-up time was 3 years. In the prematched intention-to-treat (ITT) model, SGLT2is use was associated with lower risks of dialysis (adjusted hazard ratio [aHR]: 0.21; 95% confidence interval [CI]: 0.10–0.44; *P* < 0.001), progression to macroalbuminuria (aHR: 0.86; 95% CI: 0.79–0.94; *P* = 0.001), major adverse cardiovascular events (MACE) (aHR: 0.85; 95% CI: 0.76–0.95; *P* = 0.005), acute kidney injury (AKI) (aHR: 0.68; 95% CI: 0.56–0.82; *P* < 0.001), and all-cause mortality (aHR: 0.63; 95% CI: 0.52–0.76; *P* < 0.001) compared with no SGLT2is use. After propensity score matching, multivariable analyses showed that SGLT2is use was associated with a lower risk of progression to macroalbuminuria (aHR: 0.84; 95% CI: 0.73–0.97; *P* = 0.015), and all-cause mortality (aHR: 0.40; 95% CI: 0.28–0.59; *P* < 0.001) compared with not using SGLT2is.

**Conclusion:**

This nationwide cohort study showed that SGLT2is use was associated with a significantly lower risk of progression to macroalbuminuria, and all-cause mortality compared with nonuse in patients with T2D and nonalbuminuric CKD.

## Graphical abstract

**Figure:**
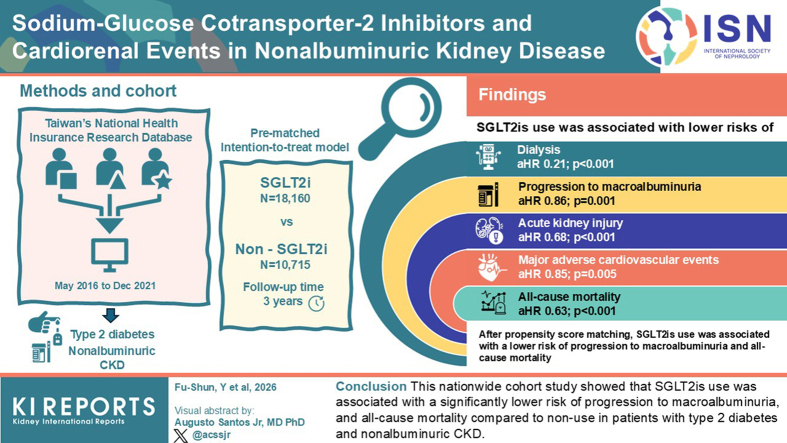

The prevalence of diabetes mellitus has escalated to epidemic levels worldwide.^1^ Concurrently, the global diabetes epidemic has led to diabetic kidney disease emerging as a significant public health and clinical issue.^2^ Furthermore, diabetic kidney disease is the leading cause of kidney failure and a major risk factor for cardiovascular disease.^2^ Based on previous research in patients with type 1 diabetes, diabetic kidney disease typically begins with microalbuminuria, progresses to macroalbuminuria, and eventually leads to a decline in renal function.^3^ Commonly observed pathological changes include thickened glomerular basement membrane, mesangial expansion, nodular glomerular sclerosis (Kimmelstiel-Wilson nodules), exudative or insudative lesions, and fibrin caps. In particular, nodular glomerular sclerosis is a hallmark of classic diabetic nephropathy.^4^^,^^5^ Importantly, recent studies have increasingly shown that both type 1 diabetes and T2D can be associated with CKD in the absence of proteinuria (urine protein-to-creatinine ratio ≥ 150 mg/g) or microalbuminuria (urine albumin-to-creatinine ratio ≧ 30mg/g).^2^ In Japan, 73% of people with a glomerular filtration rate (GFR) < 60 ml/min per 1.73 m^2^ exhibited either normoalbuminuria (52%) or microalbuminuria (21%).^6^ Similarly, the National Evaluation of the Frequency of Renal impairment cO-existing with Non–insulin- dependent diabetes mellitus study in Australia found that >80% of patients with a GFR < 60 ml/min per 1.73 m^2^ had normoalbuminuria (55%) or microalbuminuria (32%).^7^ An extended analysis of the National Health and Nutritional Examination Survey cohort, conducted 10 years after the original publication, confirmed that 52% of patients with reduced renal function had normoalbuminuria.^8^ Currently, most patients with T2D and CKD do not have microalbuminuria, and this trend is increasing.^2^^,^^9^ Although most reports suggest that nonalbuminuric CKD is associated with a slower decline in renal function and a lower incidence of cardiovascular disease than albuminuric CKD, the risk of CKD progression and cardiovascular disease compared with healthy individuals is higher.^2^^,^^9^ Therefore, the renal and cardiovascular risks in patients with nonalbuminuric CKD should not be overlooked. To date, research that examines treatment options that protect the kidneys and cardiovascular system in this patient population is limited.^2^^,^^9^

In 2016, the Empagliflozin Cardiovascular Outcome Event (EMPA-REG OUTCOME) trial reported that empagliflozin significantly reduced the progression of kidney disease and clinically relevant renal events when added to standard care, compared with a placebo.^10^ Subsequently, other renal outcome trials of SGLT2is showed that SGLT2i significantly slowed the progression of CKD, regardless of whether the patients had T2D.^11^^,^^12^ However, these renal outcome trials were mainly conducted in patients with CKD and proteinuria. Although *post hoc* and subgroup analyses of the EMPA-REG OUTCOME and Dapagliflozin Effect on Cardiovascular Events – Thrombolysis in Myocardial Infarction 58 (DECLARE-TIMI 58) trials showed that SGLT2i may reduce the risk of composite renal outcomes in patients with T2D and normoalbuminuria, the number of patients with normoalbuminuria and a GFR < 60 ml/min per 1.73 m^2^ in these studies was very small. To date, few studies have been published to evaluate whether SGLT2is can improve CKD progression and reduce cardiovascular disease risk in patients with nonalbuminuric CKD.^10^^,^^13^ Therefore, we conducted this study to compare the risk of dialysis, cardiovascular events, and mortality between SGLT2i users and nonusers with T2D and nonalbuminuric CKD.

## Methods

### Data Sources

In 1995, the Taiwanese government introduced the NHI program. Under this program, individuals pay a small premium for health care access, and most of the premium costs are covered by the government and employers. The government acts as the sole health insurance purchaser for Taiwanese citizens. By 2000, about 99% of Taiwan's 23 million residents were covered by NHI program. The NHI Research Dataset includes comprehensive health care information for insured individuals, including address, age, gender, premium levels, inpatient and outpatient diagnoses, clinical procedures, and medications.^14^ Disease diagnoses are coded using the International Classification of Diseases, 9th and 10th Clinical Modification. To ensure accuracy and appropriateness, the Health Insurance Administration conducts annual random inspections of medical records in clinics and hospitals.

This study received approval from the Institutional Review Board of the National Health Research Institutes (EC1060704-E). To protect privacy, all health care provider and patient information was encrypted and anonymized before being released. The Institutional Review Board granted a waiver of informed consent.

### Design and Procedures of the Study

**Figure 1:**
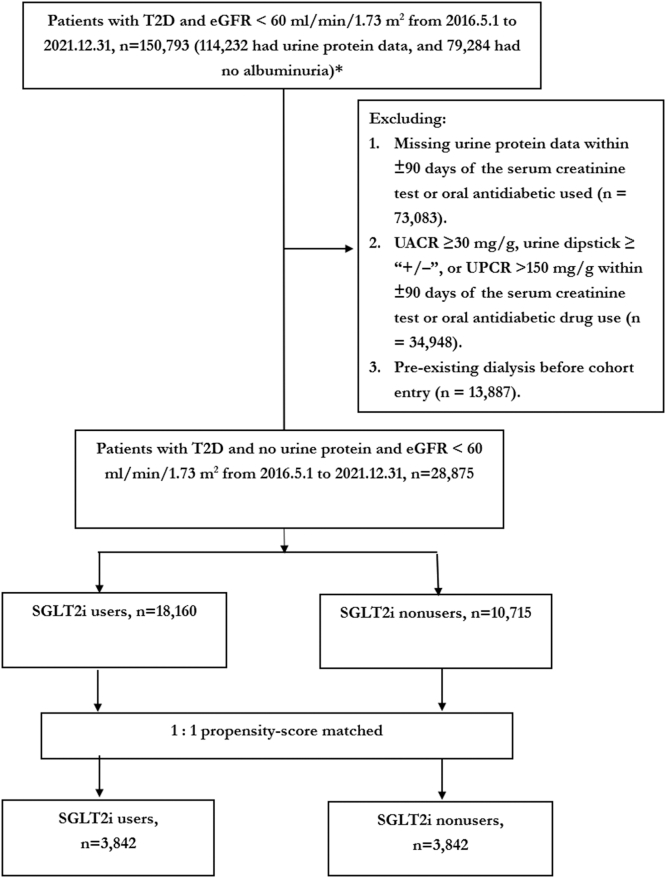
Cohort selection flowchart.∗As shown in Supplementary Table S2, among 232,420 patients with diabetes who had both albuminuria and creatinine measurements, 114,232 (232,420–118,188) had an eGFR < 60 ml/min per 1.73 m^2^, and 79,284 (167,730−88,446) had no albuminuria. eGFR, estimated glomerular filtration rate; SGLT2i, sodium-glucose cotransporter-2 inhibitors; T2D, type 2 diabetes; UACR, urine albumin-to-creatinine ratio; UPCR, urine protein-to-creatinine ratio.

Patients were defined as having T2D if they had ≥ 3 outpatient visits or 1 hospitalization with a diagnosis of T2D within 1 year (Supplementary Table S1).^15^ SGLT2is were introduced to Taiwan in May 2016. Therefore, for this study, we identified patients with T2D and nonalbuminuric CKD (urine albumin-to-creatinine ratio < 30 mg/g, urine protein-to-creatinine ratio < 150 mg/g or negative urine dipstick protein test, and eGFR < 60 ml/min per 1.73 m^2^), aged between 20 and 100 years, from May 1, 2016, to December 31, 2021 (Figure 1).^16^ The study excluded patients with missing information on age or sex, those aged < 20 years of age or those aged > 100 years at the time of initial T2D diagnosis, those with missing urine protein or blood creatinine results, those diagnosed with type 1 diabetes, and those who had ever received dialysis or kidney transplantation.

Patients who started using SGLT2is after being diagnosed with T2D and nonalbuminuric CKD were categorized as SGLT2i users. The first day of SGLT2i usage was considered the index date for this group. Those who did not use SGLT2i were placed in the control group, with their index date matched to the duration from the diagnosis of concurrent T2D and nonalbuminuric CKD to the index date of SGLT2i use.

To adjust for potential confounding factors during cohort matching and analysis (Table 1), we controlled for the following variables in the multivariable models: age; sex; obesity; and various comorbidities including hypertension, dyslipidemia, myocardial infarction, atrial fibrillation, heart failure, stroke, chronic obstruction pulmonary disease, anemia, gout, hyperkalemia, history of bleeding, peptic ulcer disease, peripheral artery disease, and cancers; and the Charlson Comorbidity Index. In addition, we included diabetes duration; medications (such as insulin, glucagon-like peptide-1 receptor agonists, metformin, sulfonylureas, meglitinides, thiazolidinediones, α-glucosidase inhibitors, dipeptidyl peptidase-4 inhibitors, angiotensin-converting enzyme inhibitors, angiotensin II receptor blockers, beta-blockers, calcium channel blockers, diuretics, fibrates, statins, aspirin, nonsteroidal antiinflammatory drugs, hypouricemic agents, and corticosteroids); laboratory values (including creatinine, estimated GFR [eGFR], CKD stages 3a to 5, glycated hemoglobin, and low-density lipoprotein cholesterol); the type of health care facility (medical center, regional hospital, district hospital, or clinic); and propensity scores (Table 1).

**Table 1:** Characteristics of SGLT2i users and nonusers in patients with type 2 diabetes and nonalbuminuric CKD

Variables	Before propensity-score matching			After propensity-score matching		
	SGLT2i nonusers	SGLT2i users	SMD	SGLT2i nonusers	SGLT2i users	SMD
	*N* = 10715	*N* = 18160		*N* = 3842	*N* = 3842	
Age groups, yrs, *n* (%)						
20–39	789 (7.4)	453 (2.5)	0.226	208 (5.4)	243 (6.3)	0.039
40–64	5735 (53.5)	7935 (43.7)	0.198	1865 (48.5)	1915 (49.8)	0.026
65–74	2566 (23.9)	6178 (34.0)	0.223	1042 (27.1)	1005 (26.2)	0.022
75+	1590 (14.8)	3590 (19.8)	0.131	714 (18.6)	676 (17.6)	0.026
Mean (SD)	60 (14.1)	64.8 (11.6)	0.370	62.4 (14.0)	61.3 (13.6)	0.078
Gender, *n* (%)						
Male	6012 (56.1)	10430 (57.4)	0.027	2125 (55.3)	2163 (56.3)	0.020
Female	4703 (43.9)	7730 (42.6)	0.027	1717 (44.7)	1679 (43.7)	0.020
Comorbidity, *n* (%)						
Myocardial infarction	1609 (15.0)	4646 (25.6)	0.265	767 (20.0)	785 (20.4)	0.012
Atrial fibrillation	247 (2.3)	633 (3.5)	0.070	106 (2.8)	135 (3.5)	0.043
Heart failure	692 (6.5)	1800 (9.9)	0.126	330 (8.6)	356 (9.3)	0.024
Dyslipidemia	6720 (62.7)	14098 (77.6)	0.330	2515 (65.5)	2828 (73.6)	0.178
Hypertension	5915 (55.2)	13104 (72.2)	0.358	2427 (63.2)	2379 (61.9)	0.026
Stroke	507 (4.7)	1073 (5.9)	0.052	196 (5.1)	209 (5.4)	0.015
Chronic obstruction pulmonary disease	829 (7.7)	1352 (7.4)	0.011	285 (7.4)	272 (7.1)	0.013
Gout	179 (1.7)	308 (1.7)	0.002	84 (2.2)	59 (1.5)	0.048
Hyperkalemia	70 (0.7)	113 (0.6)	0.004	21 (0.5)	26 (0.7)	0.017
Anemia	497 (4.6)	1026 (5.6)	0.046	209 (5.4)	200 (5.2)	0.010
Obesity	356 (3.3)	556 (3.1)	0.015	116 (3.0)	149 (3.9)	0.047
Bleeding	466 (4.3)	837 (4.6)	0.013	187 (4.9)	149 (3.9)	0.048
Peptic ulcer	910 (8.5)	1865 (10.3)	0.061	326 (8.5)	361 (9.4)	0.032
PAD	5974 (55.8)	13206 (72.7)	0.360	2439 (63.5)	2394 (62.3)	0.024
Cancer	921 (8.6)	1463 (8.1)	0.020	358 (9.3)	298 (7.8)	0.056
CCI score						
Mean (SD)	3.2 (1.7)	3.8 (1.9)	0.353	3.4 (1.8)	3.5 (1.8)	0.073
Duration of diabetes, yrs						
Mean (SD)	3.7 (2.7)	6.1 (1.9)	1.037	4.8 (2.4)	4.6 (2.2)	0.087
Prescription, *n* (%)						
Insulin	458 (4.3)	2419 (13.3)	0.324	316 (8.2)	308 (8.0)	0.008
GLP-1 RA	52 (0.5)	521 (2.9)	0.186	25 (0.7)	97 (2.5)	0.150
Metformin	9112 (85.0)	16138 (88.9)	0.114	3158 (82.2)	3364 (87.6)	0.150
Sulfonylurea	2356 (22.0)	12165 (67.0)	1.016	841 (21.9)	2219 (57.8)	0.787
Meglitinides	346 (3.2)	1363 (7.5)	0.191	134 (3.5)	239 (6.2)	0.127
α-glucosidase inhibitors	446 (4.2)	3280 (18.1)	0.453	202 (5.3)	581 (15.1)	0.331
Thiazolidinediones	403 (3.8)	4978 (27.4)	0.690	149 (3.9)	852 (22.2)	0.565
DPP-4i	3724 (34.8)	11575 (63.7)	0.606	1318 (34.3)	1999 (52.0)	0.364
ACEI	388 (3.6)	1081 (6.0)	0.109	176 (4.6)	200 (5.2)	0.029
ARB	4617 (43.1)	12002 (66.1)	0.475	1980 (51.5)	2147 (55.9)	0.087
Beta-blockers	2588 (24.2)	6627 (36.5)	0.271	1137 (29.6)	1197 (31.2)	0.034
Calcium channel blockers	2749 (25.7)	5556 (30.6)	0.110	1137 (29.6)	995 (25.9)	0.083
Diuretic	1662 (15.5)	4033 (22.2)	0.172	717 (18.7)	728 (18.9)	0.007
Statin	5756 (53.7)	13775 (75.9)	0.476	2198 (57.2)	2741 (71.3)	0.298
Fibrate	1090 (10.2)	2599 (14.3)	0.127	464 (12.1)	494 (12.9)	0.024
Aspirin	1955 (18.2)	5779 (31.8)	0.317	960 (25.0)	973 (25.3)	0.008
NSAIDs	4691 (43.8)	7882 (43.4)	0.008	1697 (44.2)	1636 (42.6)	0.032
Hypouricemic agents	1130 (10.5)	2415 (13.3)	0.085	451 (11.7)	444 (11.6)	0.006
Steroids	1116 (10.4)	1707 (9.4)	0.034	379 (9.9)	389 (10.1)	0.009
Enroll, *n* (%)						
2016	1069 (10.0)	2519 (13.9)	0.120	315 (8.2)	755 (19.7)	0.335
2017	2021 (18.9)	4223 (23.3)	0.108	596 (15.5)	968 (25.2)	0.242
2018	2069 (19.3)	3816 (21.0)	0.042	665 (17.3)	820 (21.3)	0.102
2019	1817 (17.0)	2899 (16.0)	0.027	631 (16.4)	575 (15.0)	0.040
2020	2039 (19.0)	2810 (15.5)	0.094	820 (21.3)	466 (12.1)	0.249
2021	1700 (15.9)	1893 (10.4)	0.162	815 (21.2)	258 (6.7)	0.428
Laboratory values						
Creatinine, mg/dl	4.35 (2.16)	3.71 (2.01)	0.317	4.13 (2.17)	4.13 (2.08)	< 0.001
CKD-EPI eGFR, ml/min per 1.73 m^2^	18.5 (15.1)	23.1 (17.4)	0.283	20.4 (16.4)	20.2 (16.1)	0.010
CKD stage, *n* (%)						
3a	1212 (11.3)	3534 (19.5)	0.227	553 (14.4)	561 (14.6)	0.006
3b	839 (7.8)	2083 (11.5)	0.124	373 (9.7)	341 (8.9)	0.029
4	1518 (14.2)	2518 (13.9)	0.009	532 (13.8)	546 (14.2)	0.010
5	7110 (66.4)	9994 (55.0)	0.233	2374 (61.8)	2390 (62.2)	0.009
HbA1c, % (SD)	7.83 (1.96)	8.05 (1.72)	0.118	7.81 (1.98)	7.87 (1.7)	0.030
Low-density lipoprotein-cholesterol, mg/dl (SD)	96 (33)	90 (30)	0.192	94 (32)	94 (31)	0.004
Health care facility, *n* (%)						
Medical center	2707 (25.3)	4841 (26.7)	0.032	1049 (27.3)	1061 (27.6)	0.007
Regional hospital	4772 (44.5)	8193 (45.1)	0.012	1698 (44.2)	1680 (43.7)	0.009
District hospital	2096 (19.6)	3638 (20.0)	0.012	786 (20.5)	786 (20.5)	< 0.001
Clinic	1140 (10.6)	1488 (8.2)	0.084	309 (8.0)	315 (8.2)	0.006
Propensity score (SD)	0.237 (0.220)	0.655 (0.205)	1.970	0.429 (0.307)	0.435 (0.302)	0.019

ACEI, angiotensin-converting enzyme inhibitors; ARB, angiotensin II receptor blockers; CCI, Charlson Comorbidity Index; CKD, chronic kidney disease; DPP-4i, dipeptidyl peptidase-4 inhibitors; GLP-1 RA, glucagon-like peptide-1 receptor agonists; HbA1c, glycated hemoglobin; NSAIDs, nonsteroidal antiinflammatory drugs; PAD, peripheral arterial disease; SGLT2i, sodium-glucose cotransporter-2 inhibitors; SMD: standardization mean difference.

### Key Outcomes

We assessed the incidence rate of various outcomes, including the need for dialysis, progression to macroalbuminuria (urine albumin-to-creatinine ratio ≥ 300 mg/g or urine protein 3+), admission due to anemia, MACE (a composite of hospitalization for stroke, acute myocardial infarction, or heart failure), heart failure admission, myocardial infarction admission, AKI, diabetic ketoacidosis (DKA), and all-cause mortality during the follow-up period, comparing SGLT2i users and nonusers. The diagnoses for dialysis, heart failure, stroke, myocardial infarction, anemia, AKI, and DKA were determined using International Classification of Diseases 9th and 10th Clinical Modification and procedure codes.^15^ Mortality was defined as either being discharged from a hospital with a death certificate or being discharged with a critical illness and losing NHI coverage. To evaluate the risk of these outcomes, we monitored patients from the index date until an event occurred, death, or the end of the study on December 31, 2021, whichever came first.

### Statistical Analysis

A total of 50 clinically relevant variables and propensity scores were considered as independent covariates for the analyses (Table 1). To test statistical differences, the chi-square test was used for categorical variables, and the *t* test was used for continuous variables between the study and control groups. Propensity score matching was applied to minimize the differences between SGLT2i users and nonusers. A nonparsimonious multivariable logistic regression with SGLT2i as the dependent variable was used to estimate the propensity score for each patient. The nearest neighbor algorithm, with a width of < 0.001, was employed to construct matched pairs, and standardized mean differences (SMDs) of < 0.1 were considered optimal.

Outcomes were evaluated using prematched ITT, as-treated, time-varying, and inverse probability of treatment weighting (IPTW) models. In the post–propensity score–matched cohorts, analyses were conducted using the as-treated model; therefore, patients were censored once they discontinued or switched medications. Time-to-event outcomes between SGLT2i users and nonusers were analyzed using Cox proportional hazards models. Robust sandwich standard error estimates were applied to account for the dependence introduced by propensity score matching and to ensure valid standard errors and CIs in the regression analysis. In addition, in the propensity score–matched cohort, we performed competing risk analyses for other outcomes, treating death as a competing event. The results are presented as aHRs with 95% CIs. Kaplan-Meier curves and the log-rank test were used to examine the cumulative incidences of dialysis, progression to macroalbuminuria, AKI, anemia, MACE, and survival between SGLT2i users and nonusers. Subgroup analyses were performed to assess the risk of dialysis, and all-cause mortality between SGLT2i users and nonusers, and a likelihood ratio test was conducted to identify significant interactions between SGLT2i use and factors, such as sex; age; comorbidities; medications; duration of diabetes; health care facility; biochemical tests; and CKD stages 3a, 3b, 4, and 5. A 2-tailed *P*-value < 0.05 was considered statistically significant. Analyses were performed using SAS version 9.4 (SAS Institute Inc., Cary, NC) and Stata SE version 16.1 (StataCorp, College Station, TX).

## Results

### Study Participants

Among patients with diabetes who undertook both albuminuria and creatinine testing, approximately half had CKD stage ≥3a, and 72.2% (167,730/232,420) were nonalbuminuric. Within the nonalbuminuric CKD stage 3 to 5 subgroup, stage 3 accounted for 60.8% and stages 4 and 5 for 39.2% (Supplementary Table S2). Based on GFR and albuminuria categories (Supplementary Table S3), a considerable proportion of patients with CKD stage 4 and 5 were classified as G4A1 (*n* = 7937; 3.41%) and G5A1 (*n* = 23,165; 9.97%).

A total of 28,875 patients with T2D and nonalbuminuric CKD were identified from the NHI Research Dataset. After excluding ineligible patients, there were 18,160 SGLT2i users and 10,715 SGLT2i nonusers (Figure 1). Before propensity score matching, SGLT2i users were generally older, had more comorbidities, longer durations of T2D, and were receiving more intensive medical treatment for both T2D and cardiovascular disease. In addition, they had higher glycated hemoglobin levels and better renal function compared with SGLT2i nonusers (Table 1).

After propensity score matching, 3,842 pairs of SGLT2i users and non-users were selected. The matched pairs were similar on most variables, with standardized mean differences of < 0.1. However, SGLT2i users had higher use of non-insulin antidiabetic medications and statins. The mean age of the matched SGLT2i users and non-users was 61.8 years, the mean proportion of women was 44.2%, the mean duration of diabetes was 4.7 years, the mean glycated hemoglobin was 7.84%, and the mean eGFR was 20.3 ml/min per 1.73 m^2^. Moreover, the mean proportions of CKD stages 3a, 3b, 4, and 5 were 14.6%, 9.4%, 14%, and 62%, respectively (Table 1). The mean follow-up time (SD) was 3 (1.6) years.

### Renal Outcomes

In the prematched ITT cohorts, 25 (0.137%) SGLT2i users and 19 (0.177%) SGLT2i nonusers required dialysis. The incidence rate was 0.43 versus 0.63 per 1000 person-years (Supplementary Table S4). In the postmatched cohorts, the incidence rates of dialysis between SGLT2i users and nonusers were 0.39 versus 1.19 per 1000 person-years (Table 2). The aHRs (95% CIs) for dialysis between SGLT2i users and nonusers in the prematched ITT model, the as-treated model, the IPTW model, the time-varying exposure model, and the postmatched model were 0.21 (0.10–0.44), 0.43 (0.20–0.93), 0.54 (0.17–1.74), 0.47 (0.33–0.68), and 0.45 (0.12–1.70), respectively. Compared with SGLT2i nonusers, the aHRs (95% CIs) for progression to macroalbuminuria among SGLT2i users in the prematched ITT model, the as-treated model, the IPTW model, the time-varying exposure model, and the postmatched model were 0.86 (0.79–0.94), 0.67 (0.61–0.74), 1.43 (1.24–1.65), 0.81 (0.80–0.83), and 0.84 (0.73–0.97), respectively; the aHRs (95% CIs) for anemia admission among SGLT2i users in the prematched ITT model, the as-treated model, the IPTW model, the time-varying exposure model, and the post-matched model were 0.83 (0.68–1.02), 0.79 (0.62–1.00), 0.99 (0.70–1.41), 0.92 (0.87–0.96), and 0.74 (0.54–1.03), respectively (Supplementary Table S4; Table 2).

**Table 2:** The outcome risks between matched SGLT2i users and nonusers in patients with diabetes and nonalbuminuric CKD

After propensity-score matching	SGLT2i users *N* = 3842		SGLT2i nonusers *N* = 3842		SGLT2i users vs. nonusers			
	*n*	IR	*n*	IR	cHR (95% CI)	*P*-value	aHR (95% CI)	*P*-value
Chronic dialysis	< 3	0.39	11	1.19	0.32 (0.09–1.16)	0.082	0.45 (0.12–1.70)	0.237
Chronic dialysis^a^					0.32 (0.09–1.16)	0.082	0.45 (0.12–1.70)	0.237
Progression to macroalbuminuria	324	42.16	506	58.21	0.88 (0.76–1.01)	0.072	0.84 (0.73–0.97)	0.015
Progression to macroalbuminuria^a^					0.88 (0.76–1.01)	0.072	0.84 (0.73–0.97)	0.015
Anemia admission	62	8.14	101	11.82	0.73 (0.53–1.00)	0.051	0.74 (0.54–1.03)	0.072
Anemia admission^a^					0.73 (0.53–1.00)	0.050	0.74 (0.54–1.03)	0.072
Major adverse cardiovascular events	250	33.99	297	36.16	0.96 (0.81–1.14)	0.676	0.91 (0.77–1.09)	0.307
Major adverse cardiovascular events^a^					0.96 (0.81–1.14)	0.676	0.91 (0.77–1.09)	0.307
Heart failure admission	172	23.13	179	21.27	1.10 (0.89–1.36)	0.366	1.05 (0.85–1.31)	0.627
Heart failure admission^a^					1.10 (0.89–1.36)	0.36551	1.05 (0.85–1.31)	0.627
Myocardial infarction admission	42	5.51	46	5.34	1.13 (0.74–1.72)	0.570	1.08 (0.70–1.66)	0.729
Myocardial infarction admission^a^					1.13 (0.74–1.72)	0.570	1.08 (0.70–1.66)	0.729
Diabetic ketoacidosis	138	18.39	166	19.72	0.98 (0.78–1.23)	0.890	1.01 (0.81–1.27)	0.908
Diabetic ketoacidosis^a^					0.98 (0.78–1.23)	0.890	1.01 (0.81–1.27)	0.908
Acute kidney injury	73	9.61	107	12.48	0.82 (0.61–1.11)	0.195	0.84 (0.62–1.14)	0.266
Acute kidney injury^a^					0.82 (0.61–1.11)	0.195	0.84 (0.62–1.14)	0.266
All-cause mortality	38	4.95	116	12.56	0.43 (0.30–0.63)	< 0.001	0.40 (0.28–0.59)	< 0.001

aHR, adjusted hazard ratio; cHR, crude hazard ratio; CI, confidence interval; CKD, chronic kidney disease; IR, incidence rate, per 1000 persons-years; SGLT2i, sodium-glucose cotransporter-2 inhibitors.

aHR, Model adjusted for age groups, sex, comorbidities, medications, CKD stages, duration of diabetes, and biochemical results as listed in Table 1.

a Death was treated as a competing event in the cause-specific competing risk analysis for non-fatal outcomes.

### Cardiovascular Outcomes

Compared with SGLT2i nonusers, the aHRs (95% CIs) for MACE of SGLT2i users in the prematched ITT model, the as-treated model, the IPTW model, the time-varying exposure model, and the postmatched model were 0.85 (0.76–0.95), 0.84 (0.74–0.96), 0.85 (0.70–1.04), 0.95 (0.92–0.98), and 0.91 (0.77–1.09), respectively; the aHRs (95% CIs) for heart failure admission of SGLT2i users in the prematched ITT model, the as-treated model, the IPTW model, the time-varying exposure model, and the postmatched model were 0.92 (0.80–1.06), 0.95 (0.81–1.12), 0.84 (0.66–1.07), 0.98 (0.95–1.01), and 1.05 (0.85–1.31), respectively. The aHRs (95% CIs) for myocardial infarction admission of SGLT2i users in the prematched ITT model, the as-treated model, the IPTW model, the time-varying exposure model, and the postmatched model were 0.99 (0.73–1.36), 0.96 (0.68–1.36), 0.78 (0.45–1.36), 1.01 (0.94–1.09), and 1.08 (0.70–1.66), respectively (Supplementary Table S4; Table 2).

### Safety Outcomes

Compared with SGLT2i nonusers, the aHRs (95% CIs) for AKI among SGLT2i users in the prematched ITT model, the as-treated model, the IPTW model, the time-varying exposure model, and the postmatched model were 0.68 (0.56–0.82), 0.67 (0.53–0.83), 0.84 (0.60–1.19), 0.88 (0.84–0.93), and 0.84 (0.62–1.14), respectively. The aHRs (95% CIs) for DKA among SGLT2i users in the prematched ITT model, the as-treated model, the IPTW model, the time-varying exposure model, and the postmatched model were 0.88 (0.76–1.03), 0.92 (0.77–1.09), 1.12 (0.86–1.46), 0.94 (0.91–0.98), and 1.01 (0.81–1.27), respectively. The aHRs (95% CIs) for all-cause mortality among SGLT2i users in the prematched ITT model, the as-treated model, the IPTW model, the time-varying exposure model, and the postmatched model were 0.63 (0.52–0.76), 0.34 (0.27–0.44), 1.48 (1.08–2.03), 0.69 (0.66–0.74), and 0.40 (0.28–0.59), respectively (Supplementary Table S4; Table 2).

### Competing Risk, Cumulative Incidence, and Subgroup Analyses

Because the number of deaths was relatively small, the findings from the competing risk analysis were nearly identical to those of the primary analysis. SGLT2i use remained significantly associated with a lower risk of progression to macroalbuminuria (aHR: 0.84; 95% CI: 0.73–0.97) compared with nonuse (Table 2).

**Figure 2:**
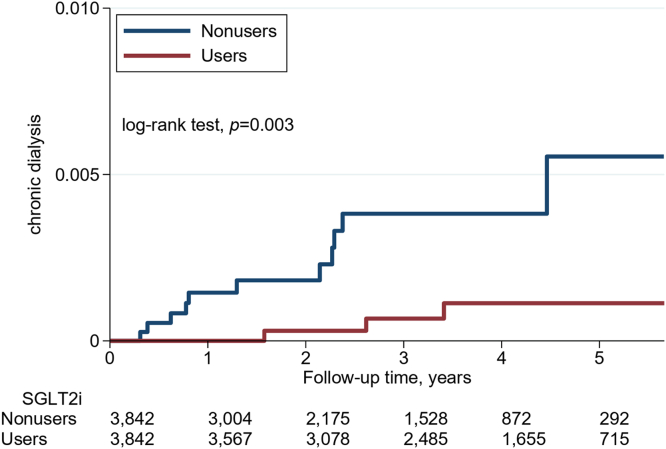
Cumulative incidence of chronic dialysis in matched SGLT2i users and nonusers. Note: The matched patients were those included after propensity-score matching, as presented in Table 1. SGLT2i, sodium-glucose cotransporter-2 inhibitors.

**Figure 3:**
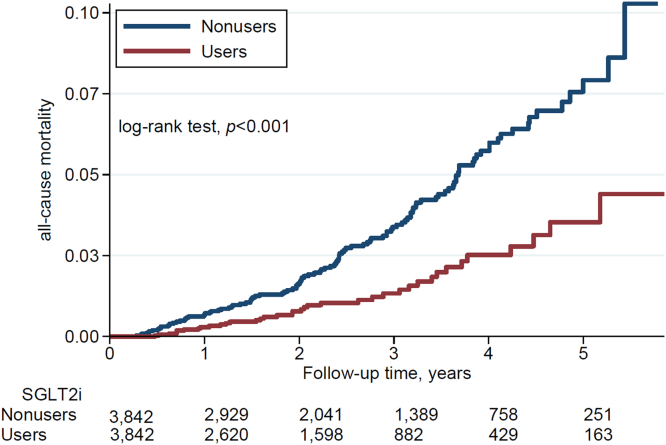
Cumulative incidence of all-cause mortality in matched SGLT2i users and nonusers. Note: The matched patients were those included after propensity-score matching, as presented in Table 1. SGLT2i, sodium-glucose cotransporter-2 inhibitors.

The matched SGLT2i users were associated with a significantly lower cumulative incidence of chronic dialysis (log-rank test, *P* = 0.003), and all-cause mortality (log-rank test, *P* < 0.001) than the matched nonusers (Figures 2 and 3).

The multivariable-adjusted subgroup analyses indicated that SGLT2i use was associated with aHRs < 1 for the risk of all-cause mortality—compared with nonuse—in several subgroups defined by age; sex; comorbidities; concomitant medications; duration of T2D; type of health care facility; and CKD stages 3b, 4, and 5 (Supplementary Tables S5 and S6). However, these subgroups had low event rates and wide CIs, resulting in nonsignificant findings. Therefore, these results should be interpreted with caution.

Subgroup analyses assessing the interaction between angiotensin II receptor blocker use and SGLT2i treatment revealed significant interactions for the outcomes of DKA (P for interaction <0.001) and all-cause mortality (P for interaction = 0.01). These findings suggest that among patients not receiving angiotensin II receptor blockers, SGLT2i therapy may confer greater reductions in the risks of DKA and death (Supplementary Table S7).

## Discussion

This nationwide cohort study showed that SGLT2i use was associated with a significantly lower risk of progression to macroalbuminuria, and all-cause mortality in the matched patients with T2D and nonalbuminuric CKD compared with SGLT2i nonusers.

Although patients with nonalbuminuric CKD experience a slower rate of decline in kidney function than those with albuminuric CKD, their rate of decline is still faster than that of the general population.^2^^,^^9^ Previous studies of renin-angiotensin system blockers on renal outcomes have been conducted in patients with proteinuria.^17^^,^^18^ Although the EMPA-REG OUTCOME and DECLARE-TIMI 58 trials included some patients with normoalbuminuria, the number of patients with normoalbuminuria and a GFR < 60 ml/min per 1.73 m^2^ was very small.^10^^,^^13^ As a result, the use of *post hoc* and subgroup analyses from these trials to assess the impact of SGLT2i on renal outcomes in patients with nonalbuminuric CKD would have been underpowered. Our research showed that SGLT2i use was associated with a significantly lower risk of dialysis among patients with nonalbuminuric CKD in both the ITT and as-treated analyses before propensity score matching. However, this association was no longer significant in the post–propensity score–matched cohorts. Moreover, because the number of chronic dialysis events in this study was relatively small, these results should be interpreted with caution.

Previous studies have shown that a small number of patients with nonalbuminuric CKD may progress to albuminuria and have an increased cardiovascular risk.^19^^,^^20^ Specifically, once proteinuria occurs, it may combine with preexisting renal dysfunction to increase the cardiovascular disease risk, accelerate the decline in renal function, and increase the risk of mortality.^2^ In contrast, our results showed that the use of SGLT2is in patients with nonalbuminuric CKD significantly reduced the risk of progression to macroalbuminuria compared to SGLT2i nonusers.

The possible mechanisms by which SGLT2is reduce the risk of progression to macroalbuminuria are as follows. (i) SGLT2i reduces intraglomerular pressure, thereby reducing shear stress on glomerular microvessels. This helps alleviate endothelial dysfunction and podocyte apoptosis, thereby mitigating glomerulosclerosis. Moreover, SGLT2i reduces proximal tubule overload, which in turn, reduces tubule atrophy and interstitial fibrosis.^21^^,^^22^ (i) SGLT2i can increase glucagon and ketone body levels, while reducing NLRP3 inflammasome activation. This leads to reduced levels of proinflammatory cytokines, resulting in less tissue inflammation and damage in patients with nonalbuminuric CKD.^22^^,^^23^ (iii) SGLT2i lowers blood glucose levels, activates sirtuin-1 expression, which reduces the production of reactive oxygen species and oxidative stress. The inhibition of oxidative stress helps prevent apoptosis in epithelial cells and slows the progression of kidney fibrosis.^24^

CKD, especially when advanced, often leads to anemia due to the reduced production of erythropoietin by renal tubules.^20^ One recent systemic review reported that SGLT2i may increase hematocrit and erythropoietin levels in patients with T2D.^22^ Pathological studies suggest that nonalbuminuric CKD is primarily characterized by tubulointerstitial fibrosis and vascular injury, which can also lead to reduced erythropoietin production and anemia.^2^^,^^25^ Our study showed that the use of SGLT2i in patients with nonalbuminuric CKD significantly reduced the risk of anemia-related admissions compared with SGLT2i nonusers. However, this association was not statistically significant in the post–propensity score–matched cohorts.

Abnormal kidney function, as measured by eGFR, is a risk factor for cardiovascular disease and related mortality.^2^^,^^6^^,^^26^ Previous studies have shown that patients with nonalbuminuric CKD have a higher risk of cardiovascular events and mortality than those without CKD.^27^, ^28^, ^29^ Normoalbuminuric CKD may be more related to macroangiopathy than microangiopathy.^2^^,^^9^ Large randomized trials have shown that SGLT2i can reduce the risk of cardiovascular disease and mortality.^30^ Our results showed that in the pre–propensity score–matched cohorts, SGLT2i use in patients with nonalbuminuric CKD was associated with lower risks of MACE and all-cause mortality compared with nonusers. In the post–propensity score–matched cohorts, SGLT2i use remained significantly associated only with a reduced risk of all-cause mortality. Moreover, subgroup analyses indicated that among patients not receiving angiotensin II receptor blockers, SGLT2i therapy might be particularly beneficial in further lowering the risk of death.

This study carries important clinical implications. Nonalbuminuric CKD accounts for a considerable proportion of patients with T2D and CKD (approximately 42.7%). Our findings indicate that SGLT2i therapy in this population may reduce the risks of progression to macroalbuminuria and all-cause mortality. These results suggest that SGLT2i treatment may confer renal and survival benefits in patients with T2D and CKD, even in the absence of albuminuria.

This study has several limitations. First, the NHI database does not include information on family history, diet, salt intake, exercise, smoking, and alcohol drinking, which could influence the results. Second, we did not regularly assess renal function and urine protein levels for each patient, so we could not observe trends of renal function changes. In addition, we lacked comprehensive imaging and pathology data, which prevented us from determining the exact cause of CKD. Third, urine protein measurement sometimes relied on a urine dipstick test when urine microalbumin or protein data were not available. This approach may introduce a degree of imprecision into the trajectories. Fourth, several concomitant medications—specifically sulfonylureas, α-glucosidase inhibitors, thiazolidinediones, dipeptidyl peptidase-4 inhibitors, and statins—were not adequately balanced between the study groups, potentially reducing the comparability between the treatment and control cohorts. Although these variables were included as covariates in the multivariable analyses to mitigate confounding, this limitation should still be acknowledged. Fifth, this study primarily focused on the Taiwanese population, so the results may not be generalizable to other ethnic groups. Finally, as a retrospective cohort study, even with careful variable adjustments, unknown or unmeasured confounding factors may be inevitable. Therefore, our results can only suggest associations rather than causality. Prospective randomized trials are needed to validate our findings.

This population-based cohort study showed that SGLT2i use was associated with a significantly lower risk of progression to macroalbuminuria, and all-cause mortality in patients with T2D and nonalbuminuric CKD than SGLT2i nonusers.

## Disclosure

All the authors declared no competing interests.

## Supplementary Material

Supplementary File (PDF) **Table S1.** Diseases and associated ICD-9th and ICD-10th codes. **Table S2.** Distribution of chronic kidney disease stages among patients with diabetes who underwent albuminuria and creatinine testing. **Table S3.** Distribution of GFR and albuminuria categories among diabetic patients tested for albuminuria and creatinine. **Table S4.** The outcome risks between pre-matched SGLT2i users and nonusers in patients with type 2 diabetes and nonalbuminuric CKD. **Table S5.** Risk of chronic dialysis in matched SGLT2i users and nonusers, stratified by variables. **Table S6.** Risk of all-cause mortality in matched SGLT2i users and nonusers, stratified by variables. **Table S7.** Comparison of outcome risks between matched SGLT2i users and nonusers, modified by concomitant use or nonuse of ARBs. STROBE Checklist.

## Footnotes

Supplementary File (PDF)
**Table S1.** Diseases and associated ICD-9th and ICD-10th codes. **Table S2.** Distribution of chronic kidney disease stages among patients with diabetes who underwent albuminuria and creatinine testing. **Table S3.** Distribution of GFR and albuminuria categories among diabetic patients tested for albuminuria and creatinine. **Table S4.** The outcome risks between pre-matched SGLT2i users and nonusers in patients with type 2 diabetes and nonalbuminuric CKD. **Table S5.** Risk of chronic dialysis in matched SGLT2i users and nonusers, stratified by variables. **Table S6.** Risk of all-cause mortality in matched SGLT2i users and nonusers, stratified by variables. **Table S7.** Comparison of outcome risks between matched SGLT2i users and nonusers, modified by concomitant use or nonuse of ARBs. STROBE Checklist.
