# Supplementary material for: Sodium-Glucose Cotransporter-2 Inhibitors and Cardiorenal Events in Nonalbuminuric Kidney Disease
Source: Kidney Int Rep. 2025 Dec 30;11(3):103756. doi: 10.1016/j.ekir.2025.103756 (PMC12860907; doi:10.1016/j.ekir.2025.103756)
Supplement: Supplementary File (PDF) — Table S1. Diseases and associated ICD-9th and ICD-10th codes. Table S2. Distribution of chronic kidney disease stages among patients with diabetes who underwent albuminuria and creatinine testing. Table S3. Distribution of GFR and albuminuria categories among diabetic patients tested for albuminuria and creatinine. Table S4. The outcome risks between pre-matched SGLT2i users and nonusers in patients with type 2 diabetes and nonalbuminuric CKD. Table S5. Risk of chronic dialysis in matched SGLT2i users and nonusers, stratified by variables. Table S6. Risk of all-cause mortality in matched SGLT2i users and nonusers, stratified by variables. Table S7. Comparison of outcome risks between matched SGLT2i users and nonusers, modified by concomitant use or nonuse of ARBs. STROBE Checklist. [file mmc1.pdf]

Supplementary Material

Sodium-Glucose Cotransporter-2 Inhibitors and Cardiorenal Events in Nonalbuminuric Kidney Disease

Fu-Shun Yen, Keong Chong, Chien-Wei Huang, James Cheng-Chung Wei, Jia-Sin Liu, Yi-Ling Wu, Chih-Ming Chen, Chii-Min Hwu, Chih-Cheng Hsu

STROBE Checklist
Supplementary Table S1. Diseases and associated ICD-9th and ICD-10th codes
Supplementary Table S2. Distribution of chronic kidney disease stages among patients with diabetes who underwent albuminuria and creatinine testing
Supplementary Table S3. Distribution of GFR and albuminuria categories among diabetic patients tested for albuminuria and creatinine
Supplementary Table S4. The outcome risks between pre-matched SGLT2i users and nonusers in patients with type 2 diabetes and nonalbuminuric CKD
Supplementary Table S5. Risk of chronic dialysis in matched SGLT2i users and nonusers, stratified by variables
Supplementary Table S6. Risk of all-cause mortality in matched SGLT2i users and nonusers, stratified by variables
Supplementary Table S7. Comparison of outcome risks between matched SGLT2i users and nonusers, modified by concomitant use or nonuse of ARB

STROBE Statement—Checklist of items that should be included in reports of ***cohort studies***

	Item No	Recommendation	Page
**Title and abstract**	1	(*a*) Indicate the study's design with a commonly used term in the title or the abstract<br>(*b*) Provide in the abstract an informative and balanced summary of what was done and what was found	1-4
**Introduction**			
Background/rationale	2	Explain the scientific background and rationale for the investigation being reported	5-6
Objectives	3	State specific objectives, including any prespecified hypotheses	6
**Methods**			
Study design	4	Present key elements of study design early in the paper	7-9
Setting	5	Describe the setting, locations, and relevant dates, including periods of recruitment, exposure, follow-up, and data collection	7
Participants	6	(*a*) Give the eligibility criteria, and the sources and methods of selection of participants. Describe methods of follow-up<br>(*b*) For matched studies, give matching criteria and number of exposed and unexposed	7-8
Variables	7	Clearly define all outcomes, exposures, predictors, potential confounders, and effect modifiers. Give diagnostic criteria, if applicable	8-9
Data sources/ measurement	8*	For each variable of interest, give sources of data and details of methods of assessment (measurement). Describe comparability of assessment methods if there is more than one group	8-9
Bias	9	Describe any efforts to address potential sources of bias	8
Study size	10	Explain how the study size was arrived at	7
Quantitative variables	11	Explain how quantitative variables were handled in the analyses. If applicable, describe which groupings were chosen and why	10
Statistical methods	12	(*a*) Describe all statistical methods, including those used to control for confounding<br>(*b*) Describe any methods used to examine subgroups and interactions<br>(*c*) Explain how missing data were addressed<br>(*d*) If applicable, explain how loss to follow-up was addressed<br>(*e*) Describe any sensitivity analyses	10
**Results**			
Participants	13*	(a) Report numbers of individuals at each stage of study—eg numbers potentially eligible, examined for eligibility, confirmed eligible, included in the study, completing follow-up, and analysed	11

		(b) Give reasons for non-participation at each stage<br>(c) Consider use of a flow diagram	
Descriptive data	14*	(a) Give characteristics of study participants (eg demographic, clinical, social) and information on exposures and potential confounders<br>(b) Indicate number of participants with missing data for each variable of interest<br>(c) Summarise follow-up time (eg, average and total amount)	11
Outcome data	15*	Report numbers of outcome events or summary measures over time	11-13
Main results	16	(*a*) Give unadjusted estimates and, if applicable, confounder-adjusted estimates and their precision (eg, 95% confidence interval). Make clear which confounders were adjusted for and why they were included<br>(*b*) Report category boundaries when continuous variables were categorized<br>(*c*) If relevant, consider translating estimates of relative risk into absolute risk for a meaningful time period	11-13
Other analyses	17	Report other analyses done—eg analyses of subgroups and interactions, and sensitivity analyses	13
**Discussion**			
Key results	18	Summarise key results with reference to study objectives	14
Limitations	19	Discuss limitations of the study, taking into account sources of potential bias or imprecision. Discuss both direction and magnitude of any potential bias	16
Interpretation	20	Give a cautious overall interpretation of results considering objectives, limitations, multiplicity of analyses, results from similar studies, and other relevant evidence	14-16
Generalisability	21	Discuss the generalisability (external validity) of the study results	16
**Other information**			
Funding	22	Give the source of funding and the role of the funders for the present study and, if applicable, for the original study on which the present article is based	17

*Give information separately for exposed and unexposed groups.

Note: An Explanation and Elaboration article discusses each checklist item and gives methodological background and published examples of transparent reporting. The STROBE checklist is best used in conjunction with this article (freely available on the Web sites of PLoS Medicine at http://www.plosmedicine.org/, Annals of Internal Medicine at http://www.annals.org/, and Epidemiology at http://www.epidem.com/). Information on the STROBE Initiative is available at http://www.strobe-statement.org.

Supplementary Table S1. Diseases and associated ICD-9th and ICD-10th codes

Disease	ICD-9-CM codes	ICD-10-CM codes
Type 2 diabetes	250.xx, except 250.1x	E11
Type 1 diabetes	250.1x	E10
Chronic kidney disease	250.4, 403-404, 585-586, 581.8, 593.9, 791.0	I12, I13, N08, N18, N19, N29, E10.2, E11.2, E13.2, N02.8, N04.7, N04.8, N18.9, N28.9, R80.8, R80.9, N18.1-N18.3, R80.0-R80.3, E10.65, E11.65.
Dialysis	V56.0, V56.8, V45.1, V45.11	Z49.31, Z49.32, Z99.2
Obesity	278.02, 783.1, V85.2, 278.00, 649.1, V77.8, V85.3, 278.01, 649.2, V45.86, V85.4	R63.5, E66.09, E66.1, E66.8, E66.9, Z13.89, E66.01, E66.2
Hypertension	401–405, A26	I10, I11, I12, I13, I15, N26
Dyslipidemia	272	E71.30, E71.31, E71.32, E71.39, E75.21, E75.22, E75.23, E75.24, E75.25, E75.29, E75.3, E75.4, E75.5, E75.6, E77, E78.0, E78.1, E78.2, E78.3, E78.4, E78.5, E78.6, E78.70, E78.71, E78.72, E78.79, E78.8, E78.9
Acute myocardial infarction	410	I21, I22
Heart failure	428	I50
Atrial fibrillation	427	I45.0, I45.1, I45.2, I45.3, I45.4, I45.5, I45.6
Stroke	430-438	G45.0, G45.1, G45.2, G45.3, G45.4, G45.8, G45.9, G46, I60, I61, I62, I63, I65, I66, I67.0, I67.1, I67.2, I67.3, I67.4, I67.5, I67.6, I67.7, I67.8, I67.9, I68, I69
Transient ischemic attack	V12.59	Z86.73
Chronic obstructive pulmonary disease	491, 492, or 496	J41, J42, J44, J43, or J44.9

Cancers	140-208	C00-C96
Gout	274, V77.5, 790.6	E79.0
Peripheral artery disease	440.0, 440.20, 440.21, 440.22, 440.23, 440.24, 440.3, 440.4, 443.9, 443.81, 443.89	I70.2, I70.92, I75.0, I73.9
Hyperkalemia	276.7	E87.5
Anemia in chronic kidney disease	285.21	D63.1
Liver cirrhosis	571.5, 571.2, 571.6	K70.2, K70.30, K70.31, K74.0, K74.1, K74.2, K74.60, K74.69, K74.3, K74.4, K74.5
Acute kidney injury	584	N17.0, N17.1, N17.2, N17.8, N17.9
Diabetic ketoacidosis	250.1	E10.10, E11.65, E11.69
Peptic ulcer diseases	531, 532, 533	K2, K26, K27, K56.6

Supplementary Table S2. Distribution of chronic kidney disease stages among patients with diabetes who underwent albuminuria and creatinine testing

	UACR, mg/g			
	**<30**	**30-300**	**> 300**	**Overall**
**CKD stage, n**				
**1 and 2**	88446	23886	5856	118188
**3a**	24950	8083	2474	35507
**3b**	23232	7880	2567	33679
**4**	7937	3438	1303	12678
**5**	23165	7316	1887	32368
**Total**	167730	50603	14087	232420

UACR, urine albumin-creatinine ratio; CKD, chronic kidney disease.

Supplementary Table S3. Distribution of GFR and albuminuria categories among diabetic patients tested for albuminuria and creatinine.

GFR /Albuminuria categories	n	Percentage	Cumulative number	Cumulative percentage
G1A1	46925	20.19	46925	20.19
G1A2	11779	5.07	58704	25.26
G1A3	2743	1.18	61447	26.44
G2A1	41521	17.86	102968	44.3
G2A2	12107	5.21	115075	49.51
G2A3	3113	1.34	118188	50.85
G3aA1	24950	10.73	143138	61.59
G3aA2	8083	3.48	151221	65.06
G3aA3	2474	1.06	153695	66.13
G3bA1	23232	10.00	176927	76.12
G3bA2	7880	3.39	184807	79.51
G3bA3	2567	1.10	187374	80.62
G4A1	7937	3.41	195311	84.03
G4A2	3438	1.48	198749	85.51
G4A3	1303	0.56	200052	86.07
G5A1	23165	9.97	223217	96.04
G5A2	7316	3.15	230533	99.19
G5A3	1887	0.81	232420	100

GFR, glomerular filtration rates; ACR, albumin-creatinine ratio.

GFR (G) categories: G1, GFR $\geqq$ 90 mL/min per 1.73 m^2; G2, GFR = 60-89 mL/min per 1.73 m^2; G3a, GFR = 45-59 mL/min per 1.73 m^2; G3b, GFR = 30-44 mL/min per 1.73 m^2; G4, GFR = 15-29 mL/min per 1.73 m^2; G5, GFR < 15 mL/min per 1.73 m^2.

Albuminuria (A) categories: A1, ACR < 30mg/g; A2, ACR = 30-300mg/g; A3, ACR > 300mg/g. Rows with a gray background—specifically the G3aA1, G3bA1, G4A1, and G5A1 categories—represent patients with non-albuminuric CKD.

Supplementary Table S4. The outcome risks between pre-matched SGLT2i users and nonusers in patients with type 2 diabetes and nonalbuminuric CKD

**Before propensity-score matching**	**SGLT2i users N=18160**		**SGLT2i nonusers N=10715**		**SGLT2i users vs. nonusers**			
	**n**	**IR**	**n**	**IR**	**cHR (95%CI)**	**p-value**	**aHR (95%CI)**	**p-value**
**Intention-to-treat design**								
Chronic dialysis	25	0.43	19	0.63	0.67 (0.37-1.22)	0.190	0.21 (0.10-0.44)	<0.001
Progression to macroalbuminuria	3595	69.62	1407	49.44	1.39 (1.31-1.48)	<0.001	0.86 (0.79-0.94)	0.001
Anemia admission	602	10.62	281	9.44	1.11 (0.97-1.28)	0.135	0.83 (0.68-1.02)	0.0732
Major adverse cardiovascular events	1872	34.48	791	27.42	1.26 (1.16-1.37)	<0.001	0.85 (0.76-0.95)	0.005
Heart failure admission	1293	23.37	488	16.59	1.42 (1.28-1.57)	<0.001	0.92 (0.80-1.06)	0.269

Myocardial infarction admission	272	4.76	103	3.43	1.38 (1.10-1.73)	0.005	0.99 (0.73-1.36)	0.964
Diabetic ketoacidosis	1075	19.37	553	18.89	1.02 (0.92-1.13)	0.673	0.88 (0.76-1.03)	0.103
Acute kidney injury	563	9.92	349	11.74	0.84 (0.73-0.96)	0.009	0.68 (0.56-0.82)	<0.001
All-cause mortality	659	11.44	303	10.03	1.10 (0.96-1.26)	0.172	0.63 (0.52-0.76)	<0.001
**As-treated design**								
Chronic dialysis	25	0.75	19	0.66	1.11 (0.60-2.02)	0.743	0.43 (0.20-0.93)	0.032
Progression to macroalbuminuria	1571	46.93	1246	44.63	1.50 (1.40-1.62)	<0.001	0.67 (0.61-0.74)	<0.001
Anemia admission	293	8.85	265	9.64	0.97 (0.82-1.15)	0.742	0.79 (0.62-1.00)	0.049
Major adverse cardiovascular events	1105	34.37	753	28.24	1.24 (1.13-1.36)	<0.001	0.84 (0.74-0.96)	0.011
Heart failure admission	769	23.65	461	16.96	1.41 (1.25-1.58)	<0.001	0.95 (0.81-1.12)	0.564

Myocardial infarction admission	163	4.9	98	3.53	1.51 (1.17-1.95)	0.001	0.96 (0.68-1.36)	0.830
Diabetic ketoacidosis	604	18.41	524	19.35	1.00 (0.89-1.13)	0.95	0.92 (0.77-1.09)	0.321
Acute kidney injury	284	8.56	333	12.11	0.76 (0.64-0.89)	0.001	0.67 (0.53-0.83)	<0.001
All-cause mortality	179	5.35	280	9.71	0.64 (0.53-0.77)	<0.001	0.34 (0.27-0.44)	<0.001
**Inverse probability of treatment weighting (IPTW)**								
Chronic dialysis	25	0.71	19	2.97	0.34 (0.15-0.78)	0.01	0.54 (0.17-1.74)	0.30
Progression to macroalbuminuria	3595	113.23	1407	236.35	1.12 (1.01-1.25)	0.04	1.43 (1.24-1.65)	<0.001
Anemia admission	602	17.28	281	44.47	0.97 (0.75-1.26)	0.81	0.99 (0.70-1.41)	0.96
Major adverse cardiovascular events	1872	56.06	791	129.59	1.07 (0.92-1.24)	0.41	0.85 (0.70-1.04)	0.11
Heart failure admission	1293	38.01	488	78.34	1.15 (0.96-1.39)	0.13	0.84 (0.66-1.07)	0.17

Myocardial infarction admission	272	7.74	103	16.15	1.16 (0.76-1.75)	0.49	0.78 (0.45-1.36)	0.39
Diabetic ketoacidosis	1075	31.53	553	88.88	1.00 (0.83-1.22)	0.96	1.12 (0.86-1.46)	0.40
Acute kidney injury	563	16.14	349	55.18	0.83 (0.64-1.06)	0.14	0.84 (0.60-1.19)	0.33
All-cause mortality	659	18.62	303	47.21	0.94 (0.74-1.21)	0.63	1.48 (1.08-2.03)	0.01
**Time-varying model**								
Chronic dialysis	.	.	.	.	0.66 (0.50-0.86)	0.003	0.47 (0.33-0.68)	<0.001
Progression to macroalbuminuria	.	.	.	.	0.92 (0.90-0.94)	<0.001	0.81 (0.80-0.83)	<0.001
Anemia admission	.	.	.	.	0.97 (0.93-1.01)	0.175	0.92 (0.87-0.96)	0.001
Major adverse cardiovascular events	.	.	.	.	1.03 (1.00-1.05)	0.036	0.95 (0.92-0.98)	<0.001
Heart failure admission	.	.	.	.	1.06 (1.03-1.09)	<0.001	0.98 (0.95-1.01)	0.226
Myocardial infarction admission	.	.	.	.	1.08 (1.01-1.15)	0.021	1.01 (0.94-1.09)	0.765

Diabetic ketoacidosis	.	.	.	.	0.96 (0.93-1.00)	0.028	0.94 (0.91-0.98)	0.001
Acute kidney injury	.	.	.	.	0.91 (0.87-0.95)	<0.001	0.88 (0.84-0.93)	<0.001
All-cause mortality		.	.	.	0.81 (0.77-0.85)	<0.001	0.69 (0.66-0.74)	<0.001

Abbreviation: SGLT2i, sodium glucose cotransporter-2 inhibitors; CKD, chronic kidney disease; ITT, intention-to-treat; IR, Incidence rate, per 1000 persons-years; cHR, crude hazard ratio; aHR, adjusted hazard ratio; CI, confidence interval.

aHR: Estimates were derived from the pre–propensity score–matched cohort shown in Table 1. Model adjusted for age groups, sex, comorbidities, medications, CKD stages, duration of diabetes, and biochemical results as listed in Table 1.

Supplementary Table S5. Risk of chronic dialysis in matched SGLT2i users and nonusers, stratified by variables

		SGLT2i users		SGLT2i nonusers		SGLT2i users vs. nonusers	
**Variables**		**n**	**IR**	**n**	**IR**	**cHR (95% CI)**	**aHR (95% CI)**
Age, years	20-39	.	.	.	1.41	0.00 (0.00- .)	. (. - .)
	40-64	.	0.42	.	0.33	0.96 (0.09-10.6)	1E13 (0.00- .)
	65-74	.	.	.	1.27	0.00 (0.00- .)	0.00 (0.00- .)
	75+	.	2.64	.	1.78	1.40 (0.25-7.79)	1.24 (0.10-16.0)
Gender	male	.	1.16	.	0.73	1.38 (0.33-5.87)	1.84 (0.38-8.92)
	female	.	.	6	1.12	0.00 (0.00- .)	0.00 (0.00- .)
Heart failure	With	.	2.4	.	0.88	2.13 (0.13-34.1)	0.02 (0.00- .)
	Without	.	0.46	10	0.91	0.51 (0.11-2.37)	0.63 (0.13-3.04)
Myocardial infarction	With	.	2.2	.	0.41	4.15 (0.38-45.8)	3381 (0.00- .)
	Without	.	0.26	10	1.03	0.26 (0.03-2.04)	0.30 (0.04-2.48)
Stroke	With	.	.	.	.	. (. - .)	. (. - .)
	Without	.	0.66	11	0.96	0.68 (0.19-2.45)	0.96 (0.25-3.69)
Hypertension	With	.	0.67	8	1.03	0.65 (0.14-3.13)	1.23 (0.21-7.22)
	Without	.	0.57	.	0.68	0.76 (0.08-7.41)	0.42 (0.01-14.1)
ARB	With	.	0.83	6	1.01	0.84 (0.17-4.27)	1.96 (0.24-15.9)
	Without	.	0.43	.	0.8	0.49 (0.06-4.27)	0.70 (0.07-6.88)
Calcium channel blockers	With	.	0.82	7	1.92	0.43 (0.05-3.51)	0.61 (0.05-7.31)
	Without	.	0.57	.	0.47	1.13 (0.20-6.21)	1.19 (0.18-8.10)
Beta-blockers	With	.	2.06	6	1.59	1.32 (0.32-5.42)	2.49 (0.47-13.2)

	Without	.	.	.	0.6	0.00 (0.00- .)	0.00 (0.00- .)
Lipid drugs	With	.	2.99	.	2.39	1.14 (0.10-12.9)	2E13 (0.00- .)
	Without	.	0.45	9	0.79	0.56 (0.12-2.64)	0.73 (0.14-3.69)
Insulin	With	.	.	6	6.31	0.00 (0.00- .)	0.00 (0.00- .)
	Without	.	0.7	.	0.45	1.50 (0.35-6.37)	2.30 (0.44-11.9)
Aspirin	With	.	0.87	.	0.95	0.76 (0.08-7.49)	8E50 (0.00- .)
	Without	.	0.56	8	0.89	0.64 (0.13-3.07)	0.87 (0.16-4.69)
Hypouricemic agents	With	.	2.02	.	2.68	0.70 (0.08-6.44)	0.00 (0.00- 498)
	Without	.	0.47	7	0.66	0.71 (0.15-3.48)	0.93 (0.18-4.95)
Steroids	With	.	.	.	0.78	0.00 (0.00- .)	11.1 (0.00- .)
	Without	.	0.7	10	0.92	0.75 (0.20-2.78)	1.25 (0.31-4.99)
NSAIDs	With	.	0.49	.	0.76	0.61 (0.07-5.51)	0.41 (0.02-7.25)
	Without	.	0.74	7	1.02	0.72 (0.15-3.54)	0.88 (0.13-5.92)
CKD stage 3b	With	.	5.54	.	2.42	2.47 (0.41-15.1)	3.53 (0.25-48.8)
	Without	.	0.23	8	0.73	0.30 (0.04-2.41)	0.33 (0.04-3.15)
CKD stage 4	With	.	.	.	.	. (. - .)	. (. - .)
	Without	.	0.72	11	1.06	0.66 (0.18-2.39)	0.96 (0.25-3.69)
CKD stage 5	With	.	0.32	8	1.09	0.28 (0.04-2.30)	0.33 (0.04-3.15)
	Without	.	1.2	.	0.62	1.95 (0.32-11.9)	3.53 (0.25-48.8)
Healthcare facility							
Medical center	With	.	0.75	.	1.5	0.50 (0.06-4.41)	0.25 (0.01-4.29)
	Without	.	0.59	6	0.68	0.82 (0.16-4.10)	1.07 (0.18-6.24)

Regional hospital	With	.	0.47	.	0.95	0.45 (0.05-3.89)	0.45 (0.04-5.34)
	Without	.	0.77	6	0.87	0.90 (0.18-4.58)	0.86 (0.14-5.28)
District hospital	With	.	1.01	.	.	9E7 (0.00- .)	18E4 (0.00- .)
	Without	.	0.53	11	1.14	0.46 (0.10-2.08)	0.62 (0.13-3.02)
Clinic	With	.	.	.	0.97	0.00 (0.00- .)	. (. - .)
	Without	.	0.67	10	0.9	0.71 (0.19-2.62)	1.12 (0.28-4.43)
Duration of diabetes, years							
0-2	With	.	0.53	.	0.17	2.29 (0.14-36.6)	0.00 (0.00- .)
	Without	.	.	.	.	0.00 (0.00- .)	1.12 (0.28-4.43)
3-5	With	.	.	.	0.69	0.00 (0.00- .)	2764 (0.00- .)
	Without	.	.	.	.	2764 (0.00- .)	1.12 (0.28-4.43)
>5	With	.	1.27	8	2.38	0.53 (0.11-2.51)	0.80 (0.14-4.57)
	Without	.	.	.	.	0.80 (0.14-4.57)	1.12 (0.28-4.43)

SGLT2i, sodium glucose cotransporter-2 inhibitors; IR, Incidence rate, per 1000 person-years; cHR, crude hazard ratio; aHR, adjusted hazard ratio; CI, confidence interval; ARB, angiotensin II receptor blockers; NSAIDs, non-steroidal anti-inflammatory drugs; CKD, chronic kidney disease.

Some cells contain no data because the number of cases was fewer than three, in accordance with National Health Insurance data privacy regulations, which restrict the release of small cell counts to prevent potential identification of individuals.

aHR: Estimates were derived from the post–propensity score–matched cohort shown in Table 1. The model was adjusted for age categories, sex, comorbidities, medications, CKD stage, diabetes duration, and the biochemical variables listed in Table 1.

Supplementary Table S6. Risk of all-cause mortality in matched SGLT2i users and nonusers, stratified by variables

		**SGLT2i users**		**SGLT2i nonusers**		**SGLT2i users vs. nonusers**	
**Variables**		**n**	**IR**	**n**	**IR**	**cHR (95% CI)**	**aHR (95% CI)**
Age, years	20-39	.	.	.	.	. (. - .)	. (. - .)
	40-64	7	2.91	20	3.33	1.05 (0.44-2.52)	0.98 (0.39-2.48)
	65-74	8	6.22	31	9.83	0.83 (0.38-1.82)	0.89 (0.40-1.99)
	75+	11	14.52	77	34.22	0.55 (0.29-1.03)	0.59 (0.30-1.13)
Gender	male	18	6.94	72	10.53	0.84 (0.50-1.43)	0.90 (0.52-1.55)
	female	8	3.72	56	10.48	0.45 (0.21-0.95)*	0.49 (0.23-1.04)
Heart failure	With	7	16.83	29	25.54	0.82 (0.35-1.90)	0.67 (0.26-1.74)
	Without	19	4.39	99	8.97	0.62 (0.38-1.02)	0.64 (0.39-1.06)
Myocardial infarction	With	7	7.72	46	18.97	0.55 (0.25-1.23)	0.66 (0.29-1.50)
	Without	19	4.95	82	8.41	0.73 (0.44-1.21)	0.77 (0.46-1.29)
Stroke	With	8	34.91	19	29.11	1.58 (0.68-3.70)	2.90 (1.02-8.25)*
	Without	18	3.99	109	9.46	0.54 (0.33-0.89)*	0.56 (0.34-0.93)*
Hypertension	With	20	6.69	102	13.12	0.65 (0.40-1.06)	0.80 (0.48-1.31)

	Without	6	3.42	26	5.91	0.71 (0.29-1.75)	0.73 (0.28-1.88)
ARB	With	16	6.62	67	11.27	0.77 (0.44-1.35)	0.86 (0.48-1.54)
	Without	10	4.29	61	9.79	0.54 (0.28-1.07)	0.59 (0.30-1.17)
Calcium channel blockers	With	11	8.97	41	11.2	0.95 (0.49-1.87)	1.41 (0.70-2.86)
	Without	15	4.26	87	10.22	0.55 (0.32-0.96)*	0.57 (0.33-1.01)
Beta-blockers	With	10	6.88	46	12.19	0.68 (0.34-1.36)	0.78 (0.38-1.59)
	Without	16	4.86	82	9.76	0.66 (0.38-1.13)	0.75 (0.43-1.31)
Lipid drugs	With	.	5.98	.	1.19	8.46 (0.71- 101)	32E6 (0.00- .)
	Without	24	5.44	127	11.2	0.61 (0.39-0.96)*	0.67 (0.43-1.05)
Insulin	With	.	6.96	18	18.78	0.51 (0.15-1.74)	0.91 (0.21-3.86)
	Without	23	5.33	110	9.81	0.68 (0.43-1.08)	0.73 (0.46-1.16)
Aspirin	With	10	8.73	42	13.31	0.89 (0.44-1.80)	0.94 (0.46-1.95)
	Without	16	4.44	86	9.53	0.58 (0.34-1.00)*	0.66 (0.38-1.15)
Hypouricemic agents	With	6	12.15	24	16	0.91 (0.37-2.28)	1.10 (0.42-2.87)
	Without	20	4.7	104	9.74	0.62 (0.38-1.01)	0.68 (0.42-1.12)

Steroids	With	.	9.04	20	15.54	0.77 (0.26-2.28)	0.98 (0.28-3.42)
	Without	22	5.11	108	9.92	0.65 (0.41-1.03)	0.71 (0.44-1.14)
NSAIDs	With	12	5.92	60	11.38	0.71 (0.38-1.32)	0.87 (0.45-1.67)
	Without	14	5.15	68	9.85	0.63 (0.35-1.13)	0.63 (0.35-1.14)
CKD stage 3b	With	6	16.62	39	31.42	0.64 (0.27-1.52)	0.84 (0.34-2.10)
	Without	20	4.56	89	8.14	0.72 (0.44-1.19)	0.74 (0.45-1.22)
CKD stage 4	With	.	6.88	17	9.51	1.10 (0.36-3.42)	3.35 (0.84-13.3)
	Without	22	5.28	111	10.68	0.61 (0.39-0.98)*	0.64 (0.40-1.03)
CKD stage 5	With	10	3.24	42	5.72	0.76 (0.38-1.53)	0.70 (0.34-1.45)
	Without	16	9.63	86	17.81	0.68 (0.39-1.17)	0.74 (0.43-1.29)
Healthcare facility							
Medical center	With	8	6.02	36	10.8	0.77 (0.35-1.68)	0.78 (0.35-1.76)
	Without	18	5.27	92	10.4	0.62 (0.37-1.04)	0.66 (0.39-1.12)
Regional hospital	With	13	6.08	55	10.45	0.69 (0.38-1.28)	0.82 (0.44-1.55)
	Without	13	4.99	73	10.56	0.63 (0.35-1.15)	0.64 (0.35-1.18)

District hospital	With	.	4.02	28	11.01	0.45 (0.16-1.31)	0.47 (0.16-1.41)
	Without	22	5.87	100	10.38	0.72 (0.45-1.15)	0.79 (0.49-1.28)
Clinic	With	.	3.53	9	8.71	0.58 (0.07-4.71)	0.33 (0.03-4.22)
	Without	25	5.6	119	10.68	0.66 (0.43-1.02)	0.74 (0.48-1.16)
Duration of diabetes, years							
0-2	With	10	5.29	35	5.93	1.27 (0.61-2.62)	1.84 (0.86-3.92)
	Without	.	.	.	.	1.84 (0.86-3.92)	0.74 (0.48-1.16)
3-5	With	.	1.56	30	10.36	0.21 (0.05-0.87)*	0.29 (0.07-1.28)
	Without	.	.	.	.	0.29 (0.07-1.28)	0.74 (0.48-1.16)
>5	With	14	8.89	63	18.65	0.57 (0.32-1.02)	0.71 (0.39-1.29)
	Without	.	.	.	.	0.71 (0.39-1.29)	0.74 (0.48-1.16)

SGLT2i, sodium glucose cotransporter-2 inhibitors; IR, Incidence rate, per 1000 person-years; cHR, crude hazard ratio; aHR, adjusted hazard ratio; CI, confidence interval; ARB, angiotensin II receptor blockers; NSAIDs, non-steroidal anti-inflammatory drugs; CKD, chronic kidney disease.

Some cells contain no data because the number of cases was fewer than three, in accordance with National Health Insurance data privacy regulations, which restrict the release of small cell counts to prevent potential identification of individuals.

aHR: Estimates were derived from the post–propensity score–matched cohort shown in Table 1. The model was adjusted for age categories, sex, comorbidities, medications, CKD stage, diabetes duration, and the biochemical variables listed in Table 1. * $p<0.05$.

Supplementary Table S7. Comparison of outcome risks between matched SGLT2i users and nonusers, modified by concomitant use or nonuse of ARB

			SGLT2i users		SGLT2i nonusers		SGLT2i users vs. nonusers		
**Outcomes**	**Variables**		**n**	**IR**	**n**	**IR**	**cHR (95% CI)**	**aHR (95% CI)**	**P for interaction**
Chronic dialysis	ARB	With	22	1.08	10	0.87	1.21 (0.57-2.57)	0.57 (0.23-1.41)	0.27
		Without	.	0.23	9	0.52	0.43 (0.12-1.61)	0.18 (0.03-0.98)	.
Progression to macroalbuminuria	ARB	With	1044	51.1	596	53.49	1.35 (1.22-1.50)	0.64 (0.56-0.73)	0.72
		Without	527	40.41	650	38.74	1.51 (1.34-1.70)	0.72 (0.61-0.84)	.
Anemia admission	ARB	With	181	8.96	120	10.96	0.88 (0.70-1.11)	0.88 (0.64-1.21)	0.98
		Without	112	8.67	145	8.76	1.03 (0.80-1.32)	0.68 (0.47-0.96)	.
Heart failure admission	ARB	With	603	30.63	298	28	1.11 (0.96-1.27)	0.97 (0.80-1.16)	0.72
		Without	166	12.94	163	9.85	1.33 (1.07-1.66)	0.83 (0.61-1.15)	.
Myocardial infarction admission	ARB	With	117	5.77	62	5.61	1.13 (0.83-1.55)	0.86 (0.56-1.30)	0.38
		Without	46	3.54	36	2.15	1.75 (1.12-2.72)	1.19 (0.63-2.24)	.
Diabetic ketoacidosis	ARB	With	405	20.26	195	18.03	1.17 (0.98-1.39)	1.19 (0.93-1.51)	<0.001
		Without	199	15.54	329	20.22	0.82 (0.69-0.98)	0.68 (0.53-0.88)	.
Acute kidney injury	ARB	With	204	10.09	162	14.83	0.71 (0.58-0.88)	0.72 (0.54-0.96)	0.13
		Without	80	6.18	171	10.32	0.66 (0.50-0.86)	0.53 (0.36-0.77)	.
All-cause mortality	ARB	With	122	5.97	117	10.19	0.67 (0.52-0.87)	0.42 (0.30-0.59)	0.01

		Without	57	4.37	163	9.39	0.55 (0.40-0.74)	0.23 (0.16-0.34)	.

SGLT2i, sodium glucose cotransporter-2 inhibitors; ARB, angiotensin II receptor blockers; IR, Incidence rate, per 1000 person-years; cHR, crude hazard ratio; aHR, adjusted hazard ratio; CI, confidence interval.

aHR: Estimates were derived from the post–propensity score–matched cohort shown in Table 1. The model was adjusted for age groups, sex, comorbidities, medications, CKD stages, duration of diabetes, and biochemical results as listed in Table 1.
